# Rare lysosomal disease registries: lessons learned over three decades of real-world evidence

**DOI:** 10.1186/s13023-022-02517-0

**Published:** 2022-10-17

**Authors:** P. K. Mistry, P. Kishnani, C. Wanner, D. Dong, J. Bender, J. L. Batista, J. Foster

**Affiliations:** 1 Department of Medicine, Yale Liver Center, Yale University School of Medicine, 333 Cedar Street, PO Box 208019, New Haven, CT 06520 USA; 2 Division of Medical Genetics, Department of Pediatrics, Duke University, Durham, USA; 3 University Hospital of Würzburg, Würzburg, Germany; 4 Global Operations and Advocacy Lead, Rare Disease Registries, Sanofi, Cambridge, MA USA; 5 Head of Global Rare Disease Registries, Sanofi, Cambridge, MA USA; 6 Epidemiology/Biostatistics, Sanofi, Cambridge, MA USA; 7 Data Management, Sanofi, Cambridge, MA USA

**Keywords:** Lysosomal storage disorders, Rare disease, Registries, Enzyme replacement therapy, Gaucher, Fabry, Mucopolysaccharidosis type I (MPS I), Pompe, Real-world data, Real-world evidence

## Abstract

Lysosomal storage disorders (LSD) are rare diseases, caused by inherited deficiencies of lysosomal enzymes/transporters, that affect 1 in 7000 to 1 in 8000 newborns. Individuals with LSDs face long diagnostic journeys during which debilitating and life-threatening events can occur. Clinical trials and classical descriptions of LSDs typically focus on common manifestations, which are not representative of the vast phenotypic heterogeneity encountered in real-world experience. Additionally, recognizing that there was a limited understanding of the natural history, disease progression, and real-world clinical outcomes of rare LSDs, a collaborative partnership was pioneered 30 years ago to address these gaps. The Rare Disease Registries (RDR) (for Gaucher, Fabry, Mucopolysaccharidosis type I, and Pompe), represent the largest observational database for these LSDs. Over the past thirty years, data from the RDRs have helped to inform scientific understanding and the development of comprehensive monitoring and treatment guidelines by creating a framework for data collection and establishing a standard of care, with an overarching goal to improve the quality of life of affected patients. Here, we highlight the history, process, and impact of the RDRs, and discuss the lessons learned and future directions.

**Supplementary Information:**

The online version contains supplementary material available at 10.1186/s13023-022-02517-0.

## Background

Rare diseases typically demonstrate heterogeneous disease manifestations, complex natural history, and variable genotype/phenotype correlations. The rarity of these disorders poses challenges in collecting and curating large cohorts; therefore, disease knowledge is often informed by small cohorts in single centers. Lysosomal storage diseases (LSD) are a prototype of rare diseases, characterized by aberrant storage/processing of a variety of substrates in tissues and organs [1, 2]. The accumulation of these substrates to toxic levels in the lysosomes results in cellular dysfunction, multi-organ injury, and the heterogeneity of clinical manifestations that underpin physical and neurological disabilities [1, 3, 4]. More than 40 LSDs are currently known and although individually rare, collectively these disorders affect 1 in 7000 to 1 in 8000 live births [3, 5, 6].

Thirty years ago, it was recognized that there was a very limited understanding of the full clinical spectrum and natural history of rare LSDs, such as Gaucher disease (GD). GD is caused by biallelic pathogenic variants in the *GBA1* gene that underlie defective lysosomal glucocerebrosidase and toxic accumulation of beta-glucosylceramide and its bioactive metabolite, glucosylsphingosine [7, 8]. In 1991, GD type 1 (GD1) was the first LSD to be treated using macrophage-targeted enzyme replacement therapy (ERT) alglucerase [9]. The International Collaborative Gaucher Group (ICGG) Gaucher Registry was launched worldwide in 1991 for patients with GD, and was open to enrollment for both treated and untreated patients.

The ICGG Gaucher Registry (ClinicalTrials.gov ID NCT00358943, 1991) was initiated in response to regulatory commitments to establish a patient base to collect real-world data (RWD) to generate real-world evidence (RWE) for effectiveness and safety of ERT, as well as assessing the natural history of the disease. Similarly, the Fabry Registry (ClinicalTrials.gov ID NCT00196742, 2001), Mucopolysaccharidosis type I (MPS I) Registry (ClinicalTrials.gov ID NCT00144794, 2003), and Pompe Registry (ClinicalTrials.gov ID NCT00231400, 2004) (hereafter referred to as ‘the Rare Disease Registries [RDRs]’) were launched as ERTs became available for these disorders.

A major advantage of the RDRs is the capacity to collect routine clinical practice data from any patient in diverse geographies compared with the narrow patient population studied in clinical trials and single centers (Table 1). As clinical trials enroll patients that meet specific inclusion/exclusion criteria, they are not representative of the wider, more heterogeneous patient population encountered in the real world; therefore, the results from clinical trials may not be generalizable to the overall patient community. For example, in the pivotal trial by Barton et al*.* that led to the approval of ERT for GD by the US Food and Drug Administration (FDA), the effectiveness of ERT in GD1 was assessed in only 12 patients [10]. Furthermore, during this pivotal trial, only plasma glucocerebroside, splenomegaly, hepatomegaly, and thrombocytopenia served as markers of the clinical response to ERT [10]. This and other clinical trials were carried out in highly selected groups of patients with GD1, which represent only ~ 10% of cases encountered in the real-world setting, where a significant proportion of patients have undergone splenectomy and have developed advanced skeletal disease and other complications [11, 12]. These reports underscore the vast range of heterogeneous clinical manifestations of GD1, such that no two patients are exactly alike even among multiple affected siblings in individual families. For example, many patients with GD1 with mild or non-classical hematovisceral manifestations may also manifest advanced skeletal complications; however, there is no correlation between classic hematovisceral manifestations and disabling bone complications such as bone crises, bone pain, or fragility fractures [13]. Moreover, some potentially life-threatening complications of GD1, such as myeloma and pulmonary arterial hypertension, can occur in patients with low disease severity as defined by classical criteria [14].

**Table 1:** Observational registries versus clinical trials

Characteristics	Registries	Clinical studies
Purpose	Real-world observations	Controlled experiments
Duration	Indefinite	Finite
Inclusion criteria	General	Specific
Data collection	Voluntary	Required
Visits	Per medical practice	Per protocol
Analytical methods	Epidemiology	Biostatistics
Disease characteristics	Cross-sectional, longitudinal	Per protocol
Treatment outcomes	Long-term effectiveness	Efficacy and safety
Applicability	Broad patient populations	Per protocol

Over the past 30 years, the RDRs have contributed to delineating disease heterogeneity, regional disease patterns, genotype/phenotype correlations, clinical practice similarities/differences, treatment responses across the entire spectrum of the diseases, and assessing clinical outcomes of therapy. The overarching objective of the RDRs is to provide a mechanism to accurately and consistently gather long-term RWD, increase the understanding of GD, Fabry disease (FD), MPS I, and Pompe disease (PD), and provide evidence to support the optimal management and treatment of patients worldwide. Here we highlight the history, process, and impact of the RDRs, including the lessons that have been learned, and discuss future directions.

## How the Rare Disease Registries are set up and operate

### Overview of the Rare Disease Registries

The RDRs are voluntary studies designed to collect observational data from routine clinical and laboratory assessments related to the onset, progression, and outcomes of GD, FD, MPS I and PD, derived from the global patient population. All patient and physician information entered in the RDRs database remains confidential in compliance with international standards and national regulations, as well as local policies. In addition, the RDRs comply with all local rules and regulations in participating countries and participating sites, including approvals of regulatory documents by an institutional review board (IRB) or ethics committee (EC) and/or Ministry of Health (MoH) as required. The infrastructure of the registries is supported by a third party to maintain the electronic data capture application and clinical database. The management and administration of the RDR programs by the sponsor, Sanofi, includes global operations and monitoring, data management, statistical programming, and epidemiology and biostatistics teams. Since the establishment of the RDRs, regional and international boards of advisors have been critically important. These boards of advisors are comprised of independent physicians with expertise in the care of patients with GD, FD, MPS I, and/or PD, and serve as liaisons between the RDRs and the respective medical and rare disease communities, by providing scientific oversight and direction for the RDRs. Physicians from the RDR Board of Advisors also participated in the development of the recommended schedules of assessments (RSA) to establish the standard of care for patients, and to facilitate consistent and comprehensive patient evaluations throughout the medical community. In 2019, the Rare Disease Registries Patient Council was established to engage the patient community as important stakeholders in the use of Registry data to generate RWE and to add patients’ perspectives on matters including how the data are disseminated into the wider community. The initiative to include patient representation in RDRs is in keeping with the Obama administration’s Patient-Centered Outcomes Research Institute guidelines [15].

### Patient eligibility and enrollment

Any patient with a confirmed diagnosis of GD, FD, MPS I, or PD, regardless of disease severity or treatment status, can be enrolled into the respective RDR by their treating physician through the web-based electronic data capture (EDC) system and reporting platform (RegistryNXT!), which is a convenient way for physicians to participate in the RDRs. The only inclusion criterion for enrollment in the respective RDR is a confirmed diagnosis via biochemical evidence of a deficiency in enzyme activity and/or disease-causing genetic variant(s).

Patients complete and sign the Patient Informed Consent and Authorization form which is IRB/EC/MoH approved to comply with local rules and regulations. To ensure anonymity, a unique RDR number is issued to each patient. At enrollment, demographic data, the date of confirmatory diagnosis via enzyme and/or DNA analysis, and initiation of primary therapy for the LSD, if applicable, are entered.

### Data collection and analysis

The RDRs aim to balance the need to capture a broad dataset with the necessary burden of data collection, while also supporting individual patient and physician needs. For example, RegistryNXT! provides individual patient clinical summaries (PCS) in real time. Each PCS contains a summary of longitudinal data for individual patients and can be viewed in a dashboard or downloaded as a report for use by patients and clinicians.

To ensure clinically meaningful data are collected, the RDRs collect data based on the standard of care of each patient and provide RSAs within the protocols. The RSAs incorporate the core signs and symptoms related to the disease that are assessed to monitor clinical onset, progression, and outcomes over the lifelong course of the disease. However, physicians who directly manage the patients determine the appropriate frequency and type of clinical assessments specific to each patient’s needs.

The RDRs are designed to collect both natural history and outcomes data. Since, at the time of enrollment, patients will be at various stages in their disease course and medical care, both retrospective and prospective data collection are recommended to promote data uniformity among RDR patients. Collected data include, but are not limited to, patient enrollment, demographic data, patient diagnosis, medical history and follow-up clinical assessments, exams, image evaluations, and patient-reported outcomes (PROs) that can be routinely evaluated, as well as treatment regimens. The submitted data are reviewed and audited electronically for missing, incomplete, or discrepant information.

### Continual evolution of data collection

The RDRs made an early transition in 2001 to a digital platform by switching from paper case report forms (CRFs) to EDC starting in 2001. In 2009, a CRF standardization project was conducted across all four RDRs to implement industry standards for data collection as the RDRs transitioned to a web-based data collection and reporting system (RegistryNXT!) in 2011. In addition, it is recognized that, as our understanding of a disease continuously increases, it is important to evolve and remove endpoints that are redundant/do not reflect the current outcome measures, thus accommodating critical analysis of new data and growing awareness of different evidence needs [16]. Since 2017, RDR CRFs further evolved by both removing assessments that were not available for data entry and adding new assessments. Examples include the collection of malignancy and gammopathy data in GD, movement disorders related to Parkinson’s disease symptoms in GD and FD, and COVID-19 in all RDRs. In addition, specific biomarkers are important to collect as they are discovered and become part of real-world clinical care. For example, there is growing evidence that globotriaosylsphingosine (lyso-Gb3), the deacylated form of globotriaosylceramide which accumulates in FD, could be a significant risk factor associated with important clinical events in FD and therefore a valuable biomarker of disease progression [16]. However, whether treatment-related amelioration of lyso-Gb3 levels is associated with improved long-term outcomes needs to be established [16]. Similarly, in GD, serum glucosylsphingosine (lyso-Gb1, lyso-GL1) was recently added following its extensive validation in the clinic as a biomarker of functional significance in various manifestations of GD [17, 18]. In addition, it is crucial to understand the data that are being collected in standard practice and assess what data are or are not relevant to real-world care. This necessitates regular reviews to ensure all relevant data points are being captured and may lead to the removal of data that may no longer be clinically relevant.

To better understand patient experiences and preferences, quality of life (QoL) measures and PROs are becoming increasingly important in rare diseases [19]. These measures are particularly important to help ensure the outcomes that matter to patients are captured in rare disease research and are ultimately used in clinical practice [19]. Moreover, the regulatory authorities, FDA, and European Medicines Agency (EMA), acknowledge that the patients’ perspectives are important during the drug development and approval process [20]. In addition, Health Technology Assessment (HTA) agencies also value the submission of PRO data when evaluating pharmacotherapies or medical technologies. As patients are intended to be the beneficiaries of health innovations, inclusion of patient experiences and views are vital in the HTA process [19].

## Achievements

**Fig. 1:**
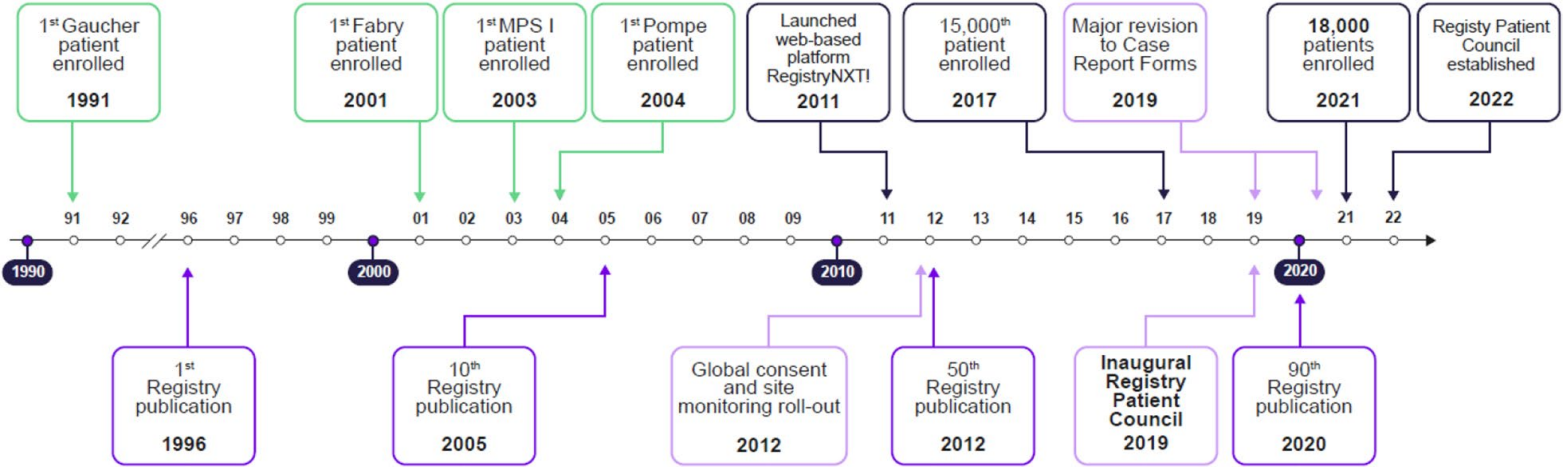
Timeline of Gaucher, Fabry, MPS I, and Pompe Registry milestones

**Fig. 2:**
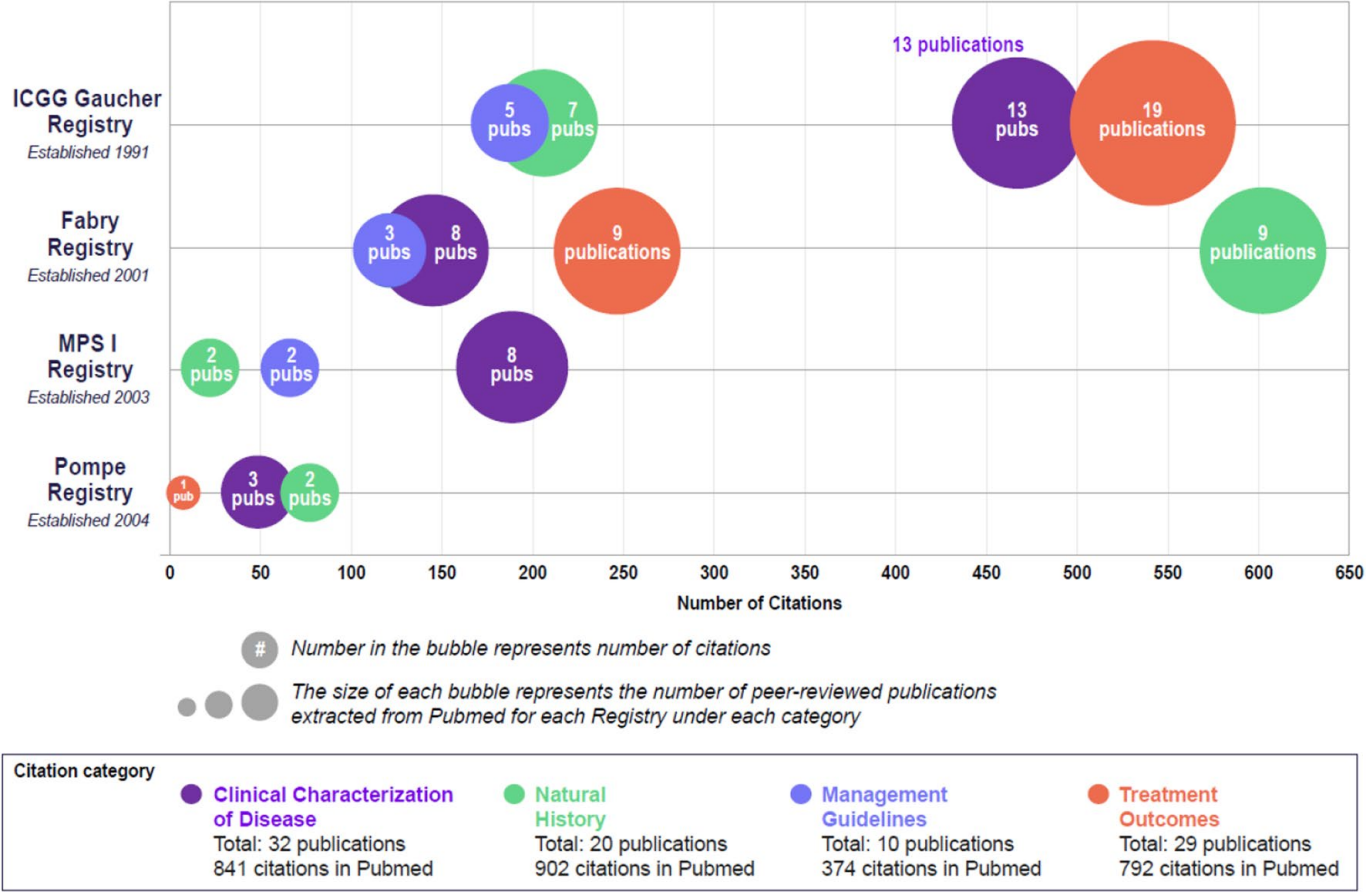
Impact of Rare Disease Registries publications

The RDRs have served the global rare disease community over the last 30 years. With more than 18,000 patients enrolled so far across 68 countries, and with the help of over 1200 healthcare professionals, the RDRs have expanded globally, capturing 716,837 and 235,593 collective person-years from birth and diagnosis, respectively, to last follow-up (Table 2). With sustained commitment to evidence generation, the RDRs have achieved a number of milestones. This is underscored by the progression from the ICGG Gaucher Registry's first patient enrollment in 1991, to a total of 18,000 patients enrolled in RDRs in 2021 (Fig. 1). More than 90 peer-reviewed manuscripts have been published that have advanced the field of research for GD, FD, MPS I, and PD (see the Additional file 1: Appendix for a list of the most cited publications on each disease). Evidence from the RDRs has informed the medical community on clinical characterization of disease, natural history, management guidelines, and treatment outcomes for each disease (Fig. 2).

**Table 2:** Total number of patients and person-years in the Rare Disease Registries

Registry	Year Registry was established	Current data			Person-years					
		Total countries*	Total Registry sites**	Total patients	Total person-years from birth to last follow-up†		Total person-years from diagnosis to last follow-up‡		Total person-years from treatment initiation to last follow-up§	
					Person-years	N	Person-years	N	Person-years	N
ICGG Gaucher Registry	1991	64	278	6872	266,543	6844	112,115	6481	67,470	5595
Fabry Registry	2001	47	243	7930	344,445	7897	78,220	7267	38,523	5017
MPS I Registry	2003	41	144	1325	18,598	1323	13,497	1297	10,086	1176
Pompe Registry	2004	47	240	2467	87,251	2463	21,761	2405	13,510	2210
Total			805	18,594	716,837	18,527	235,593	17,450	129,589	13,798

All Data as of February 2022

*Includes currently and historically active countries/regions

**Includes currently active sites where at least one patient is enrolled

^†^Data are shown for patients with non-missing dates of birth and last follow-up

^‡^Data are shown for patients with non-missing dates of diagnosis and last follow-up

^§^Data are shown for ever-treated patients with non-missing dates of treatment initiation and last follow-up

MPS I, Mucopolysaccharidosis type I

The RDRs have increased the understanding of the natural history of rare disease over time [21, 22]. Data from the ICGG Gaucher Registry have revealed GD to be a system-wide disorder, beyond the macrophage system [22, 23]. Examples include increased risk of malignancies and Parkinson’s disease in GD1, which underscore unmet needs in GD1. Epidemiological data captured by the RDRs are dramatically changing our understanding of the diseases caused by variations in *GBA*, through RWE generation [24]. First, it was discovered that the most common variant around the world is *L444P* (p.Leu483Pro), not *N370S* (p.Asn409Ser). Homozygosity for *L444P* (p.Leu483Pro) variant leads to type 2 or type 3 GD. Moreover, patients with GD1 are at a > 20-fold higher risk than the general population for developing Parkinson’s disease/Lewy body dementia, with the risk varying depending on the variant, i.e., the risk is higher for *L444P* (p.Leu483Pro) compared with *N370S* (p.Asn409Ser) [25–27]. Data from the RDRs have contributed to literature on the vast clinical spectrum of neurodegenerative diseases seen in GD [25–28].

Similarly, the Fabry Registry has transformed our understanding of disease outcomes and the impact of FD on female patients. Using Fabry Registry data, Patel et al*.* reported major cardiovascular events in approximately 5% of the Fabry Registry patients before ERT or among patients who never received therapy, indicating that patients with FD should be monitored for possible cardiovascular risk factors [29]. Females with heterozygous variations in the *GLA* gene were believed to be asymptomatic carriers due to a normal allele synthesizing α-galactosidase A enzyme [30]. The trial by Eng et al*.* evaluated the safety and clinical efficacy of the ERT agalsidase-beta for patients with FD [31]. However, the trial included only 56 male and two female participants. Lessons from the Fabry Registry have revealed that females with FD have a significant risk for major organ involvement and decreased QoL attributed to unequal inactivation of the X chromosome [32]. As a result, the Fabry Registry has made a systematic effort to enroll all females with FD regardless of symptomology. Female patients with FD should be routinely monitored and treated accordingly to improve their QoL and long-term clinical outcomes.

The RDRs have collectively captured 129,589 person-years of primary treatment information, which has been essential to understanding treatment-related outcomes for patients and has provided an extended view, beyond the scope of clinical trials, of long-term treatment-related outcomes (Table 2). Using Gaucher Registry data, Weinreb et al*.* reported that imiglucerase treatment sustains clinical improvements for 20 years in GD1 non-splenectomized and splenectomized patients [33]. Additionally, Hopkin et al*.* found that patients with FD experiencing severe clinical events while on ERT with agalsidase-beta had more advanced organ involvement at baseline compared with patients without such events, and that patients who initiated ERT at a younger age may experience less residual risk of on-ERT events [34]. Based on data from the Pompe Registry, Stockton et al*.* examined respiratory muscle function among late-onset PD (LOPD) patients during ERT with alglucosidase-alfa and found forced vital capacity is preserved during long-term ERT. This is key as, in LOPD, respiratory muscle dysfunction and failure are sources of significant morbidity and mortality [35]. Another important lesson learned from the RDRs is that efforts to improve early diagnosis in attenuated MPS I are needed [36]. Based on MPS I Registry data, Giugliani et al. concluded that, although the median age at diagnosis has not improved for individuals with attenuated MPS I, the time to treatment initiation after diagnosis has improved in the last 15 years [36].

Historically, patients with rare diseases have been underserved because of the challenges associated with smaller patient populations. Patients often experience a significant diagnostic odyssey, which may involve consultations with multiple non-specialist and specialist healthcare professionals, and misdiagnoses that ultimately delay initiation of therapy. The RDRs provide a larger sample size than would otherwise be available for epidemiological and clinical research for rare disease, which will ultimately benefit the patients. As an example, the ICGG Gaucher Registry showed that maximal impact of reducing the risk of avascular necrosis in GD1 is achieved by initiation of treatment within 2 years of diagnosis [37].

**Fig. 3:**
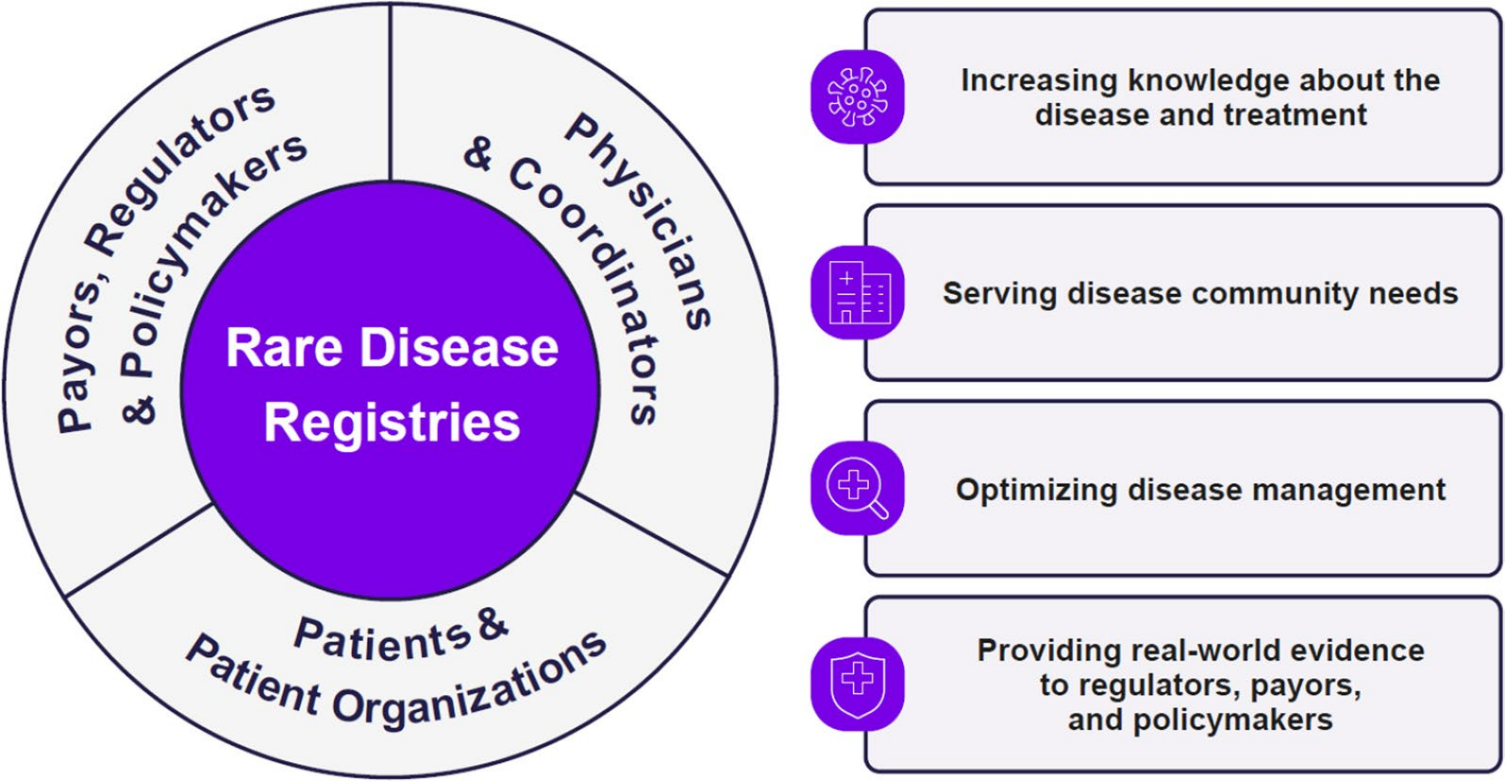
Key stakeholders

Through the years, the RDRs have been instrumental in multistakeholder engagement between the medical community, patients and patient organizations, payors, regulators, and policy makers (Fig. 3). Collaboration between stakeholders is critical for the progress made in research publications, treatment guidelines, label expansions, government reimbursement, patient care, drug approvals, post-marketing commitments, and healthcare policy. For example, survival data from the Pompe Registry supported the Secretary’s Advisory Committee on Heritable Disorders in Newborns and Children recommendation to the US Secretary of Health and Human Services to include PD in the Recommended Uniform Screening Panel in the United States [38].

Healthcare professionals support RWE dissemination through carefully designed studies and peer-reviewed manuscripts, which are increasingly utilized by regulators and HTA agencies. Specifically, the data from the RDRs are used not only to fulfill regulatory commitments and in reimbursement decisions, but also provide a better understanding of unmet needs and the heterogeneity of rare diseases, and the effectiveness and long-term safety of treatment (Fig. 2). For example, Wanner et al*.* demonstrated that urinary protein excretion is strongly associated with renal disease progression in patients enrolled in the Fabry Registry [39]. Additionally, to further understand disease progression in MPS I, Viskcohil et al*.* constructed sex- and age-specific estimated length/height and head circumference growth curves in untreated individuals as this information could provide a foundation for understanding the pathogenesis of skeletal disease in MPS I [40].

## Challenges leading to new opportunities

### Data collection and analysis

Despite the enormous contributions of RDRs thus far, there are challenges associated with analyzing registry data. First, it is important to ensure a high standard of evidence generation. Standards of traditional evidence-based medicine that are applied in highly prevalent clinical conditions are often not feasible for rare diseases. This is due to the limited knowledge on the natural history of the disease, small patient populations, and disease heterogeneity, including age of onset, responsiveness to treatment, and disease progression. These considerations underscore a need for a standardized set of outcomes for rare diseases. Some challenges can be surmounted by an ongoing multistakeholder dialogue on RWE generation [41]. For example, variant data analysis was a challenge in the Pompe Registry. Variants can be entered in different ways due to lack of standardization, variable nomenclature, letter spacing, or software-directed automatic use of upper and lower case. Extensive reviews and consultations with RDR sites were conducted to standardize the nomenclature to allow for more efficient analysis and interpretation of results [42]. Therefore, agreeing upon an accepted nomenclature and codification for rare diseases through ongoing dialogue with stakeholders is important for compiling a taxonomy of sub-populations of rare diseases.

As the RDRs are non-interventional and designed to collect RWD during routine clinical practice, it is important to note that the clinical assessments performed for each patient may differ depending on disease severity, as well as standards of care and resources available in different countries. In addition, the methods and techniques used for clinical assessment are not geographically universal. Moreover, the multisystemic nature of each disease means patients typically undergo multiple clinical and biochemical assessments and are seen by several physicians who may not be familiar with the RDRs. Therefore, the collection of uniform data for all patients can be difficult and data analysis challenging. Challenges include selecting appropriate QoL and PRO measures and ensuring these are captured consistently, collecting data on biomarkers, obtaining long-term follow-up data, and assuring data quality. To help participating clinics increase data completeness, RDR personnel offer training in data entry to participating physicians and their staff to maximize quality control of data entry.

Moreover, baseline data are not available for all patients because of the voluntary, non-interventional nature of the RDRs, and because patients may begin treatment many years before entry into the RDRs. Due to this non-interventional nature, physicians are also not obligated to enter data or answer queries in the database on discrepant or missing data. As a result, it can be difficult to assess natural history or long-term treatment effectiveness based on available data in the database. The RDRs identified the challenges of baseline data entry and implemented clear baseline data entry definitions for untreated and treated patients. The definitions were added to all forms in the database as a reminder during the data entry process.

A significant limitation of the RDRs, common for all registries, is incomplete data capture. With time, as the value proposition of the RDRs becomes more widely appreciated by stakeholders, this limitation is being addressed with greater data capture by individual sites. In the meantime, approaches such as propensity scoring and case control study designs have been utilized effectively to harness the RDRs’ data [43, 44]. In GD, the long-standing controversy of dosing versus response was effectively addressed using propensity scoring [43]. A model for future exploration to address this specific research question is to conduct focused data collections in sites with large numbers of patients that have a track record of complete data capture. This approach was recently utilized in a comprehensive study investigating the type of malignancies and the natural history of gammopathy in GD [45].

The increasing availability of high-quality longitudinal data in the RDRs over the past decades has brought with it an evolution of the statistical methods applied to rare disease studies. Analyses in the early years were largely limited to descriptive statistics and were often a cross-sectional view of the data, such as patient characteristics prior to treatment initiation [46–50]. As follow-up time has accrued in an increasing number of patients and thousands of repeated measures and clinical outcomes have been entered into the database, more sophisticated methods have been applied, such as multivariable logistic regression [13, 51–53]], mixed effects modeling [23, 35, 54–56], and Cox proportional hazards regression [57, 58]].

### Cost and resources

Significant cost and resources, such as maintaining the electronic infrastructure, data analysis, interpretation, and the reporting and dissemination of important findings to professional communities, are required to run a registry for 30 years. The collection of long-term follow-up data for rare diseases is critical for fulfilling the RDRs’ objectives; however, a unique challenge they encounter is how to encourage patient retention and minimize loss to follow-up. As such, the RDRs need to devote sufficient effort to patient and physician retention.

Publicly funded registries have been set up over the years; however, they have faced challenges regarding cost and resources. The European Union (EU) Committee of Experts on Rare Diseases was established to improve the quality of care for patients with rare diseases [59]. This committee has outlined recommendations on rare disease patient registration and data collection. However, registries can face the issues of unstable funding, unclear stakeholder roles, predominantly paper-based data collection, and insufficient data dissemination [59]. In contrast, the RDRs have been a success due to the long-term commitment toward their development and operation, from their early foundations established by Genzyme through to their ongoing support by Sanofi and continued dedication of legacy RDR team members and healthcare professionals.

### Digital innovations

Due to the heterogeneous nature of rare diseases, it is critical to learn from individual experiences and aggregate these learnings across the rare disease population to identify common themes in disease progression, management, and treatment. Thus, the RDRs have a unique challenge to cater to individual needs as well as learning from collective experiences that will lead to better patient management [60]. To develop sustainable and useful digital health solutions to address this duality, the RDRs utilize an innovative web-based, mobile-friendly, electronic data collection and reporting platform called RegistryNXT!. RegistryNXT! allows for the automation of user accounts, and the self-service eLearning capability reduces the training burden on operational site teams. RegistryNXT! also provides real-time data reporting back to the Registry sites using dynamic visualizations and filtering options, as well as a data extraction tool for sites to download their own data and access a library of Registry documents. As mentioned above, the RDRs evolved early on from a paper-based to an electronic system, allowing for quicker data entry, improved consistency, and clean-up of data discrepancies. Soon, patients will be able to enter their own data through electronic patient-reported outcomes (ePROs) and provide consent via electronic consent forms, providing them with the opportunity for more active participation in the registries.

## Future direction and continued commitment to stakeholders

While the RDRs have a 30-year history of sustained effort across the four LSDs, GD, FD, MPS I, and PD, it is important to acknowledge that the broader RWD landscape continues to evolve as different stakeholders endorse different models of disease registries, including academic, government, and patient-led registries. Across this RWD/E ecosystem with stakeholders including patients and patient advocacy groups, the medical community, healthcare authorities, and HTA agencies, the RDRs continue to evolve and make important contributions. Periodic, critical evaluation with key stakeholders is vital to ensure that the objectives are being met, and that the RDRs can adapt to changes in the clinical landscape, such as changes in treatment practice or the introduction of new therapies, and continue to collect relevant data. The RDRs governance and operations will benefit from adaptability and rapid incorporation of new knowledge to ensure the data that are captured reflect current patient routine care in different countries, as well as new developments in our understanding and management of GD, FD, MPS I, and PD.

Ongoing analysis of data from the RDRs also allows for regular assessment of the evolving landscape of RWE generation, to ensure that the current and potential partnerships are optimally structured to produce evidence of the highest relevance and impact. For example, following diagnosis of PD through newborn screening, the decision on initiation and timing of ERT for patients with LOPD can be challenging. To track the disease state in individual patients it is important to have adequate, ongoing follow-ups and assessments for disease progression. However, because numerous tests are needed at variable frequencies for monitoring, evidence-based guidelines for a standardized approach across centers were required [61]. In addition, the MPS I and Pompe Registries have implemented optional consents for family linkage to connect related individuals’ data for future analyses.

One of the limitations of registries is that there is lack of data sharing and interoperability between rare disease registries. Furthermore, only aggregated data from the RDRs can be shared with requesters after a rigorous request process. In the future, the RDRs could adopt data sharing policies to allow other researchers to access and combine data from across different Registries. For example, an independent researcher could use the data from several different Registries to assess incidence of a particular complication for informing protocol development for a new study.

It is important to acknowledge that different models of registries that include patient, patient advocacy groups, government, academic, and industry-supported models are important to ensure registries provide optimum benefit to the rare disease community. Patient-led registries are key because they recognize the need for patient engagement in monitoring and understanding long-term outcomes of rare diseases. Patients and patient association-owned registries are a critical part of evidence generation and are key partners, owners, and drivers in the evolving landscape of RWD.

The data captured by the RDRs are used by regulatory bodies, including the FDA and EMA, to make regulatory decisions. Due to the value of the evidence generated by registries in general for the FDA and EMA, the EMA has set up initiatives to make better use of existing registries. To address some challenges faced by regulators and pharmaceutical companies such as harmonized protocols, scientific methods, and data structure, the EMA seeks to create an EU-wide framework on patient registries to facilitate collaboration between medicine regulators and pharmaceutical companies [62, 63]. Considering the continued commitment to opening new RDR sites worldwide, this collaboration will be key for the successful operation and continuous evolution of such programs.

Due to the evolution of science and health technology and the popularization of personalized health concepts, wearable devices may play a great role in the future of healthcare. Wearable devices, sensors, and mobile applications can assist in constant monitoring of patients for safety and efficacy of treatment [64]. For example, a pilot study by Donald et al*.* successfully demonstrated the use of wearable technology paired with a mobile phone app to monitor physical activity as a surrogate measure of disease activity/severity. This study found patients with GD1 performed higher intensity activity in a 30-min period compared with patients with type 2 and 3 GD [65]]. In the future, the RDRs could incorporate data from wearables, providing answers to research questions in timeframes previously thought unfeasible. Currently, the RDRs are working on creating patient portals and ePROs, which is an important first step in giving patients access to personal health information from anywhere with an internet connection.

## Conclusion

Early identification, correct diagnosis, and effective long-term management of patients with rare diseases is a global issue. The RDRs are a driver of data and evidence generation that have evolved to contribute to an increased knowledge and understanding of rare diseases. RDR data leading to the generation of RWE is paramount for improving decision making about rare diseases within the healthcare multistakeholder ecosystem, from HTAs to routine clinical practice, with the common aim of improving patients’ and their caregivers’ lives.

Data from the RDRs provide a foundation for the rare disease community to continue to address unmet needs and support research into new and existing therapies, even after a treatment has been proven to be well tolerated and effective through randomized clinical trials, to inform both the medical and patient/caregiver community.

The RDRs continue to operate beyond the need to fulfill regulatory requirements by collecting data and generating evidence in an environment of high unmet medical need. The growth of web-based patient communities and advances in digital health and technology, may be integrated into RDR data collection in the future, as community building across geographical boundaries continues to become simpler. As the active patient community continues to grow, as well as the access to patient data, knowledge of rare diseases will increase and, with it, improved understanding of patient management and long-term clinical outcomes.

## Supplementary Information

**Additional file 1.** Impact of rare disease registries publications.

## Data Availability

The datasets generated and analyzed during the current study are not publicly available due to patient privacy and confidentiality. Anonymized data can be made available upon reasonable request.
