# Supplementary material for: Rare lysosomal disease registries: lessons learned over three decades of real-world evidence
Source: Orphanet J Rare Dis. 2022 Oct 17;17:362. doi: 10.1186/s13023-022-02517-0 (PMC9573793; doi:10.1186/s13023-022-02517-0)
Supplement: Supplementary file 1 — Additional file 1. Impact of rare disease registries publications. [file 13023_2022_2517_MOESM1_ESM.docx]

# Impact of Rare Disease Registries Publications

## List of most cited publications per disease

| **Registry** | **Citation category** | **Author** | **Title** | **Journal and year** |
| --- | --- | --- | --- | --- |
| **ICGG Gaucher Registry (Established 1991)** | Clinical Characterization of Disease | Mistry PK, et al. | Transformation in pretreatment manifestations of Gaucher disease type 1 during two decades of alglucerase/imiglucerase enzyme replacement therapy in the International Collaborative Gaucher Group (ICGG) Registry. | *Am J Hematol.* 2017. |
|  |  | Camelo JS, et al. | Gaucher disease type 1 in the skeleton: review of Latin America. | *Coluna/Columna*. 2006. |
|  |  | Grabowski G, et al. | Gaucher disease types 1 and 3: phenotypic characterization of large populations from the ICGG Gaucher Registry. | *Am J Hematol.* 2015. |
|  |  | Drelichman G, et al. | Gaucher disease in Argentina: report from the International Collaborative Gaucher Group (ICGG) Gaucher Registry and the Argentinian Group for Diagnosis and Treatment of Gaucher disease. | *Hematología.* 2013. |
|  |  | Drelichman G, et al. | Gaucher disease in Latin America. A report from the Gaucher Disease International Registry and the Latin American Group for Gaucher Disease. | *Medicina (B Aires).* 2012 |
|  |  | Tylki-Szymañska A, et al. | Neuronopathic Gaucher disease: demographic and clinical features of 131 patients enrolled in the International Collaborative Gaucher Group Neurological Outcomes Subregistry. | *J Inherit Metab Dis.* 2010. |
|  |  | Fairley C, et al. | Phenotypic heterogeneity of N370S homozygotes with type I Gaucher disease: an analysis of 798 patients from the ICGG Gaucher registry. | *J Inherit Metab Dis.* 2008. |
|  |  | Sobreira E, et al. | Phenotypic and genotypic heterogeneity in Gaucher disease type 1: a comparison between Brazil and the rest-of-the-world. | *Mol Genet Metab.* 2007. |
|  |  | Kaplan P, et al. | The clinical and demographic characteristics of nonneuronopathic Gaucher disease in 887 children at diagnosis. | *Arch Pediatr Adolesc Med.* 2006. |
|  |  | Gabrowski G, et al. | Gaucher disease: phenotypic and genetic variation. | Book chapter in: *The Online Metabolic and Molecular Bases of Inherited Disease.* 2019. |
|  |  | Wenstrup RJ, et al. | Skeletal aspects of Gaucher disease: a review. | *Br J Radiol.* 2002. |
|  |  | Charrow J, et al. | The Gaucher registry: demographics and disease characteristics of 1698 patients with Gaucher disease. | *Arch Intern Med.* 2000. |
|  |  | Sevittz H, et al. | Baseline characteristics of 32 patients with Gaucher disease who were treated with imiglucerase: South African data from the International Collaborative Gaucher Group (ICGG) Gaucher Registry. | *S Afr Med J.* 2022. |
|  | Natural History | Mistry PK, et al. | Understanding the natural history of Gaucher disease. | *Am J Hematol*. 2015. |
|  |  | Cox TM, et al. | Gaucher disease and comorbidities: B-cell malignancy and Parkinsonism. | *Am J Hematol.* 2015. |
|  |  | Khan A, et al. | Risk factors for fractures and avascular osteonecrosis in type 1 Gaucher disease: a study from the International Collaborative Gaucher Group (ICGG) Gaucher Registry. | *J Bone Miner Res.* 2012. |
|  |  | Cole JA, et al. | Reducing selection bias in case-control studies from rare disease registries. | *Orphanet J Rare Dis.* 2011. |
|  |  | Rosenbloom B, et al. | The incidence of Parkinsonism in patients with type 1 Gaucher disease: data from the ICGG Gaucher Registry. | *Blood Cells Mol Dis.* 2011. |
|  |  | Weinreb NJ, et al. | Life expectancy in Gaucher disease type 1. | *Am J Hematol.* 2008. |
|  |  | Rosenbloom BE, et al. | Gaucher disease and cancer incidence: a study from the Gaucher Registry. | *Blood.* 2005. |
|  | Management Guidelines | Deegan P, et al. | The International Collaborative Gaucher Group GRAF (Gaucher Risk Assessment for Fracture) score: a composite risk score for assessing adult fracture risk in imiglucerase-treated Gaucher disease type 1 patients. | *Orphanet J Rare Dis.* 2021. |
|  |  | Pastores GM, et al. | Therapeutic goals in the treatment of Gaucher disease. | *Semin Hematol.* 2004. |
|  |  | Weinreb NJ, et al. | Gaucher disease type 1: revised recommendations on evaluations and monitoring for adult patients. | *Semin Hematol.* 2004. |
|  |  | Charrow J, et al. | Enzyme replacement therapy and monitoring for children with type 1 Gaucher disease: consensus recommendations. | *J Pediatr.* 2004. |
|  |  | Charrow J, et al. | Gaucher disease: recommendations on diagnosis, evaluation, and monitoring. | *Arch Intern Med.* 1998. |
|  | Treatment Outcomes | Weinreb NJ, et al. | Gaucher disease type 1 patients from the ICGG Gaucher Registry sustain initial clinical improvements during twenty years of imiglucerase treatment. | *Mol Genet Metab.* 2021. |
|  |  | Mistry PK, et al. | Real-world effectiveness of eliglustat in treatment-naïve and switch patients enrolled in the International Collaborative Gaucher Group Gaucher Registry. | *Am J Hematol.* 2020. |
|  |  | El-Beshlawy A, et al. | Long-term hematological, visceral, and growth outcomes in children with Gaucher disease type 3 treated with imiglucerase in the International Collaborative Gaucher Group Gaucher Registry. | *Mol Genet Metab.* 2017. |
|  |  | Ibrahim J, et al. | Clinical response to eliglustat in treatment-naïve patients with Gaucher disease type 1: post-hoc comparison to imiglucerase-treated patients enrolled in the International Collaborative Gaucher Group Gaucher Registry. | *Mol Genet Metab Rep.* 2016. |
|  |  | Charrow J, Scott RC. | Long-term treatment outcomes in Gaucher disease. | *Am J Hematol.* 2015. |
|  |  | Camelo JS, et al. | Long-term effect of imiglucerase in Latin American children with Gaucher disease type 1: lessons from the International Collaborative Gaucher Group Gaucher Registry. | *BMC Hematology.* 2014. |
|  |  | Weinreb N, et al. | Long-term clinical outcomes in type 1 Gaucher disease following 10 years of imiglucerase treatment. | *J Inher Metab Dis.* 2013. |
|  |  | Hollak CE, et al. | Characteristics of type I Gaucher disease associated with persistent thrombocytopenia after treatment with imiglucerase for 4-5 years. | *Br J Haematol.* 2012. |
|  |  | Mistry PK, et al. | Osteopenia in Gaucher disease develops early in life: response to imiglucerase enzyme therapy in children, adolescents and adults. | *Blood Cells Mol Dis.* 2011. |
|  |  | Mistry PK, et al. | Timing of initiation of enzyme replacement therapy after diagnosis of type 1 Gaucher disease: effect on incidence of avascular necrosis. | *Br J Haematol.* 2009. |
|  |  | Grabowski GA, et al. | Dose-response relationships for enzyme replacement therapy with imiglucerase/alglucerase in patients with Gaucher disease type 1. | *Genet Med.* 2009. |
|  |  | Andersson H, et al. | Eight-year clinical outcomes of long-term enzyme replacement therapy for 884 children with Gaucher disease type 1. | *Pediatrics.* 2008 |
|  |  | Weinreb N, et al. | A benchmark analysis of the achievement of therapeutic goals for type 1 Gaucher disease patients treated with imiglucerase. | *Am J Hematol.* 2008. |
|  |  | Charrow J, et al. | The effect of enzyme replacement therapy on bone crisis and bone pain in patients with type 1 Gaucher disease. | *Clin Genet.* 2007. |
|  |  | Wenstrup RJ, et al. | Effect of enzyme replacement therapy with imiglucerase on BMD in type 1 Gaucher disease. | *J Bone Miner Res.* 2007. |
|  |  | Andersson HC, et al. | Individualization of long-term enzyme replacement therapy for Gaucher disease. | *Genet Med.* 2005. |
|  |  | Poll LW, et al. | Response of Gaucher bone disease to enzyme replacement therapy. | *Br J Radiol.* 2002. |
|  |  | Weinreb NJ, et al. | Effectiveness of enzyme replacement therapy in 1028 patients with type 1 Gaucher disease after 2 to 5 years of treatment: a report from the Gaucher Registry. | *Am J Med.* 2002 |
|  |  | Kaplan P, et al. | Acceleration of retarded growth in children with Gaucher disease after treatment with alglucerase. | *J Pediatr.* 1996. |
| **Fabry Registry (Established 2001)** | Clinical Characterization of Disease | Germain DP, et al. | Use of a rare disease registry for establishing phenotypic classification of previously unassigned GLA variants: a consensus classification system by a multispecialty Fabry disease genotype-phenotype workgroup. | *J Med Genet.* 2020. |
|  |  | Germain DP, et al. | Phenotypic characteristics of the p.Asn215Ser (p.N215S) GLA mutation in male and female Fabry patients: a multicenter Fabry Registry study. | *Mol Genet Genomic Med.* 2018. |
|  |  | Villalobos J, et al. | Fabry disease in Latin America: data from the Fabry Registry. | *JIMD Rep.* 2013. |
|  |  | Politei JM, et al. | Fabry disease in Argentina: an evaluation of patients enrolled in the Fabry Registry. | *Int J Clin Pract.* 2013. |
|  |  | Martins AM, et al. | Demographic characterization of Brazilian patients enrolled in the Fabry Registry. | *Genet Mol Res.* 2013. |
|  |  | Politei JM, et al. | New concepts of the natural history, evolution and treatment, related to the findings of Fabry Registry. | *Rev Nefrol Diál Traspl.* 2009. |
|  |  | Eng CM, et al. | Fabry disease: baseline medical characteristics of a cohort of 1765 men and female in the Fabry Registry. | *J Inherit Metab Dis.* 2007. |
|  |  | Giannini EH, et al. | A validated disease severity scoring system for Fabry disease. | *Mol Genet Metab.* 2010. |
|  | Natural history | Martins AM, et al. | The clinical profiles of female patients with Fabry disease in Latin America: a Fabry Registry analysis of natural history data from 169 patients based on enzyme replacement therapy status. | *JIMD Rep.* 2019. |
|  |  | Patel MR, et al. | Cardiovascular events in patients with Fabry disease: natural history data from the Fabry registry. | *J Am Coll Cardiol.* 2011. |
|  |  | Wanner C, et al. | Prognostic indicators of renal disease progression in adults with Fabry disease: natural history data from the Fabry Registry. | *Clin J Am Soc Nephrol.* 2010. |
|  |  | Ortiz A, et al. | End-stage renal disease in patients with Fabry disease: natural history data from the Fabry Registry. | *Nephrol Dial Transplant.* 2010. |
|  |  | Waldek S, et al. | Life expectancy and cause of death in males and females with Fabry disease: findings from the Fabry Registry. | *Genet Med.* 2009. |
|  |  | Sims K, et al. | Stroke in Fabry disease frequently occurs before diagnosis and in the absence of other clinical events: natural history data from the Fabry Registry. | *Stroke*. 2009. |
|  |  | Wilcox WR, et al. | Females with Fabry disease frequently have major organ involvement: lessons from the Fabry Registry. | *Mol Genet Metab.* 2008. |
|  |  | Ortiz A, et al. | Nephropathy in males and females with Fabry disease: cross-sectional description of patients before treatment with enzyme replacement therapy. | *Nephrol Dial Transpl.* 2008. |
|  |  | Hopkin RJ, et al. | Characterization of Fabry disease in 352 pediatric patients in the Fabry Registry. | *Pediatr Res.* 2008. |
|  | Management Guidelines | Hopkin RJ, et al. | The management and treatment of children with Fabry disease: a United States-based perspective. | *Mol Genet Metab.* 2016. |
|  |  | Eng CM, et al. | Fabry disease: guidelines for the evaluation and management of multi-organ system involvement. | *Genet Med.* 2006. |
|  |  | Ortiz A, et al. | Fabry disease revisited: management and treatment recommendations for adult patients. | *Mol Genet Metab.* 2018. |
|  | Treatment Outcomes | Hopkin RJ, et al. | Improvement of gastrointestinal symptoms in a significant proportion of male patients with classic Fabry disease treated with agalsidase beta: a Fabry Registry analysis stratified by phenotype. | *Mol Genet Metab Rep.* 2020. |
|  |  | Wanner C, et al. | Cardiomyopathy and kidney function in agalsidase beta treated female Fabry patients: a pre‑treatment vs. post-treatment analysis. | *ESC Heart Fail.* 2020. |
|  |  | Wilcox WR, et al. | Improvement of Fabry disease-related gastrointestinal symptoms in a significant proportion of female patients treated with agalsidase beta: data from the Fabry Registry. | *JIMD Rep.* 2018. |
|  |  | Hopkin RJ, et al. | Risk factors for severe clinical events in male and female patients with Fabry disease treated with agalsidase beta enzyme replacement therapy: data from the Fabry Registry. | *Mol Genet Metab.* 2016. |
|  |  | Ortiz A, et al. | Time to treatment benefit for adult patients with Fabry disease receiving agalsidase β: data from the Fabry Registry. | *J Med Genet.* 2016. |
|  |  | Germain DP, et al. | Ten-year outcome of enzyme replacement therapy with agalsidase beta in patients with Fabry disease. | *J Med Genet.* 2015. |
|  |  | Germain DP, et al. | Analysis of left ventricular mass in untreated men and in men treated with agalsidase-β: data from the Fabry Registry. | *Genet Med.* 2013. |
|  |  | Warnock DG, et al. | Renal outcomes of agalsidase beta treatment for Fabry disease: role of proteinuria and timing of treatment initiation: findings from the Fabry Registry. | *Nephrol Dial Transplant.* 2012. |
|  |  | Watt T, et al. | Agalsidase beta treatment is associated with improved quality of life in patients with Fabry disease: findings from the Fabry Registry. | *Genet Med.* 2010. |
| **MPS I Registry (Established 2003)** | Clinical Characterization of Disease | Giugliani R, et al. | Improvement in time to treatment, but not time to diagnosis, in patients with mucopolysaccharidosis type I. | *Arch Dis Child.* 2021. |
|  |  | Clarke LA, et al. | Genotype-phenotype relationships in mucopolysaccharidosis type I: insights from the International MPS I Registry. | *Clin Genet.* 2019. |
|  |  | Arn P, et al. | Airway-related symptoms and surgeries in patients with mucopolysaccharidosis I. | *Ann Otol Rhinol Laryngol.* 2015. |
|  |  | D’Aco K, et al. | Diagnosis and treatment trends in mucopolysaccharidosis I: findings from the MPS I Registry. | *Eur J Pediatr.* 2012. |
|  |  | Muñoz-Rojas MV, et al. | Clinical manifestations and treatment of mucopolysaccharidosis type I patients in Latin America as compared with the rest of the world. | *J Inherit Metab Dis.* 2011. |
|  |  | Thomas JA, et al. | Childhood onset of Scheie syndrome, the attenuated form of mucopolysaccharidosis I. | *J Inherit Metab Dis.* 2010. |
|  |  | Arn P, et al. | Characterization of surgical procedures in patients with mucopolysaccharidosis type I: findings from the MPS I Registry. | *J Pediatr.* 2009. |
|  |  | Pastores GM, et al. | The MPS I registry: design, methodology, and early findings of a global disease registry for monitoring patients with mucopolysaccharidosis type I. | *Mol Genet Metab.* 2007. |
|  | Natural History | Viskochil D, et al. | Growth patterns for untreated individuals with MPS I: report from the International MPS I Registry. | *Am J Med Genet.* 2019. |
|  |  | Beck M, et al. | The natural history of MPS I: global perspectives from the MPS I Registry. | *Genet Med.* 2014. |
|  | Management  Guidelines | Viskochil D, et al. | Carpal tunnel syndrome in mucopolysaccharidosis I: a registry-based cohort study. | *Dev Med Child Neurol.* 2017. |
|  |  | Arn P, et al. | High rate of postoperative mortality in patients with mucopolysaccharidosis I: findings from the MPS I Registry. | *J Pediatr Surg.* 2012. |
| **Pompe Registry (Established 2004)** | Clinical Characterization of Disease | Reuser AJJ, et al. | GAA variants and phenotypes among 1,079 patients with Pompe disease: Data from the Pompe Registry. | *Hum Mutat.* 2019. |
|  |  | Zhao Y, et al. | Characteristics of Pompe disease in China: a report from the Pompe registry. | *Orphanet J of Rare Diseases.* 2019. |
|  |  | Byrne B, et al. | Pompe disease: design, methodology, and early findings from the Pompe Registry. | *Mol Genet Metab.* 2011. |
|  | Natural History | Kishnani P, et al. | Timing of diagnosis of patients with Pompe disease: data from the Pompe Registry. | *Am J Med Genet A.* 2013. |
|  |  | Roberts M, et al. | The prevalence and impact of scoliosis in Pompe disease: lessons learned from the Pompe Registry. | *Mol Genet Metab.* 2011. |
|  | Treatment Outcomes | Stockton DW, et al. | Respiratory function during enzyme replacement therapy in late-onset Pompe disease: longitudinal course, prognostic factors, and the impact of time from diagnosis to treatment start. | *J Neurol.* 2020. |
